# New Jersey

**Published:** 1896-04

**Authors:** 


*New Jersey.* A bill has been introduced in the State Legislature to establish a State Board of Animal Industry, to consist of the President and Secretary of the Board of Agriculture and five persons appointed by the President, and $6500 annually to be appropriated for the payment for diseased animals slaughtered. Expenses of the Board to be paid by the State. The chief object is to stamp out tuberculosis.

